# Research on Influencing Factors of Satisfaction with the Use of Public Health Internet Platform: Evidence from Ding Xiang Doctor (DXY) Internet Medical Platform

**DOI:** 10.3390/ijerph20032276

**Published:** 2023-01-27

**Authors:** Yanlong Guo, Lan Zu, Denghang Chen, Han Zhang

**Affiliations:** 1 Social Innovation Design Research Centre, Anhui University, Hefei 203106, China; 2 Department of Science and Technology Communication, University of Science and Technology of China, Hefei 203106, China; 3 College of Environmental Science and Engineering, Ocean University of China, Qingdao 266100, China

**Keywords:** public health, Internet health care, user satisfaction, comprehensive evaluation

## Abstract

With the rapid development of Internet information technology, Internet medical platforms are gradually entering daily life. Especially after the outbreak of the COVID-19 pandemic, it becomes very difficult for patients to go out for medical treatment, and the Internet medical platform plays an important role. The study of the use and influencing factors of Internet medical platforms has become a new topic. In this study, evidence from the Chinese Internet medical platform Ding Xiang Doctor(DXY) is combined with an integrated approach of hierarchical analysis and the entropy value method to construct evaluation indexes and questionnaires from four dimensions of perceived quality, perceived value, user trust, and user involvement to analyze the factors influencing users’ satisfaction with Internet medical platforms. The questionnaires were distributed online, and 556 questionnaires were distributed from June to August 2022; 520 questionnaires were collected; the questionnaires’ recovery rate was 93.53%; after excluding some invalid questionnaires, 424 questionnaires remained; the questionnaire efficiency was 81.54%; the Cronbach coefficient was 0.978; the KMO(Kaiser-Meyer-Olkin) value was 0.977; and the reliability performance was good. The study concluded that: (1) Users pay more attention to the content of perceived value, including the cost of time, economy, expense, and effort spent, and emphasize the degree of personal benefit. (2) Users are less satisfied with the information accessibility, design aesthetics, information timeliness, information comprehensiveness, and classification clarity of the DXY platform. (3) Users pay most attention to the protection of personal privacy by the platform side in the dimension of perceived value. (4) Users’ trust in the platform is relatively high, their willingness to use the platform in the future is strong, and the dimensions of online interactive discussion, willingness to pay, and paid services are highly recognized.

## 1. Introduction

The outbreak of the COVID-19 pandemic has accelerated the process of consumer acceptance of online medical care. By providing online health assessment, health guidance, health education, consultation guidance, continuous disease follow-up, psychological counseling, and other medical consultation services, the Internet medical platform has met the public’s daily demand for simple and scientific care, relieved the burden on offline medical resources, and also reduced population concentration and the risk of cross infection, which has been called the “second battlefield” in the fight against the epidemic.

At the same time, China’s regulatory stance on “Internet healthcare” is becoming increasingly clear, with relevant guidance documents and policy details being issued one after another. In February 2020, the Chinese Health and Welfare Commission issued the Notice on Strengthening Information Technology to Support the Prevention and Control of the Novel Coronavirus Infection Pneumonia Epidemic [1] and the Notice on Improving Internet Medical Consultation Services in the Prevention and Control of the Epidemic [2], which mentioned that the advantages of “Internet medicine” should be used to provide the people with high-quality and convenient medical consultation services. In March of the same year, the China Medical Insurance Bureau and the China Health Insurance Commission issued the Guidance on Promoting “Internet+” Medical Insurance Services all through the Prevention and Control of the Novel Coronavirus Pneumonia Epidemic [3], which proposed that Internet clinical services should be included in the scope of clinical insurance payment. In April, China’s Development and Reform Commission and the Central Internet Information Office issued “On the Promotion of “On the Cloud with the Data to Empower Intelligence” Action to Promote the New Economy Development Implementation Plan” [4], proposed to explore the promotion of Internet medical insurance’s first consultation and appointment triage system in the field of health to carry out the Internet medical insurance settlement, medical insurance standards, online sales of drugs, graded treatment, remote consultation, multipoint practice, and family doctor and so on. With the policy dividend, the future of the Internet medical industry is promising.

The trend of internalization of China’s medical and health industry is obvious, and a number of online health community practice applications have emerged with DXY and Chun Yu Doctor as typical representatives. However, through a network survey among many Internet medical and health information service platforms, that there is no shortage of problems, such as poor service quality, poor security of user disease privacy, and homogenization of medical information, was found. Therefore, this paper constructs a quality evaluation index system of an Internet medical and health information service platform, comprehensively considers the maturity of Internet medical platform providers, and provides references for improving the service quality of Internet medical platforms. 

Ding Xiang Doctor (DXY) is a medical website developed by the Lilac team, which holds the professional license of Internet hospital. It is a one-stop service platform that integrates consultation, prescription, payment and medicine delivery, online follow-up, and regular consultation. According to DXY’s records, its platform has more than 5.5 million expert users, among which 1.47 million are registered doctors, including 71% of doctors in China, making it the largest scientific connector and expert provider company in China’s digital field. As the largest Internet medical service platform in China [5], DXY’s user experience and satisfaction scores are representative of research on public health Internet platforms.

## 2. Literature Review

### 2.1. Research on Internet Health Platform

The proliferation of the Internet has had a good effect on the quality of medical services. It was reported that Internet telemedicine use increased 10-fold in the United States in 2020 [6]. As COVID-19 is highly contagious, Internet medical platforms have become an important tool for medical professionals and patients to maintain a safe social distance [6,7,8,9,10]. The convenience of an Internet medical platform not only enables patients to receive treatment in a timely manner and avoid worsening conditions due to delays, but also improves access to high-quality care for patients in remote areas [9,10]. While public health Internet platforms provide convenience to users, they additionally supply customers with a massive number of overloaded facts, and there are nevertheless some issues. For example, transgender people face issues such as platform censorship, disinformation, hate speech, and lack of flagging inaccurate content when searching for health information online [11]. It is difficult to guarantee the privacy of users using the Internet health platform [12]. Internet health care was chosen more for convenience than accuracy or authority [13].

In addition, the impact of network penetration on the consultation price of physicians in specialized hospitals is greater than that of physicians in general hospitals [14]. Lu et al. found that online services of public health Internet platforms complement offline services (outpatient visits) and that improved ratings could lead to a relative increase in physician visits [15]. Physicians offering online health consultations can effectively increase the value of patient-submitted reviews [16]. The above studies suggest that public health Internet platforms can bring positive value to both users and health care providers and have good prospects for development, but there are still many issues, such as information accuracy, comprehensiveness, and user privacy.

### 2.2. Research Related to User Satisfaction and User Perception

Satisfaction refers to a top feeling that the user’s feeling about a product or carrier exceeds the anticipated expectation in the system of or after using it. Satisfaction affects users’ willingness to use and utilization behavior and is integral for operators to enhance carrier niceness and extend product benefits. Through literature research, it is determined that students regularly use expectation affirmation theory, and various factors to discover the elements that influence personal satisfaction in the fields of e-commerce and tourism [17]. For example, Fornel et al. observed that personal pride in advertising and marketing is positively and significantly influenced by the use of personal expectations, perceived fantasy, and perceived value [18]; Vega-Vazquez used numerical modeling to confirm the beneficial relationship between consumer enjoyment and product quality value [19]. Kuo et al. confirmed through surveys and analysis that supplier satisfaction and buyer loyalty have a positive effect on customer satisfaction. In addition, the correlation between buyer satisfaction and buyer loyalty is more desirable when customers discover high service value [20].

User perception refers to the user’s comprehensive feelings about product functions and service effects, which are generated through various senses and thoughts in the service context. User perception is influenced by internal and external factors, such as the user himself, the service provider, the service content, and the service environment, which will determine the user’s satisfaction and loyalty to the product. Harvey proposed the APEC model, in which A is aesthetic sense, P is practical effectiveness, E is emotional perception, and C is cognitive model [21]; Yoon argued that user perception consists of three parts: product visualization factors, interaction, and combination [22]; Kubat proposed that user perception mainly consists of three parts: user psychology, product performance, and content perception [23]; studies have shown that doctors’ speaking speed has a positive impact on patient satisfaction in online medical services, but this vocal feature has a substitutive effect [24]. Analysis by Jin et al. showed that perceived threat has a poor effect on trust, customer satisfaction, and customer loyalty, and confidence has a good effect on customer pride and customer loyalty [25]. Patients with mental illness are more likely to trust online treatment than face-to-face communication with doctors [26]. With the aging of the population, there is an insurmountable gap among the elderly in the digital information age [27]. The improvement of the Internet medical platform can facilitate the online treatment of the elderly. In addition, the use of the Internet can also directly reduce the degree of depression among rural elderly people [28]. Integrating applicable research on person delight by means of scholars and combining the characteristics of public health Internet platforms and user characteristics, this paper selects perceived quality and perceived value as the main factors affecting users’ satisfaction of DXY, and selects user trust and user commitment to reflect users’ satisfaction with the platform.

### 2.3. Related Research on Evaluation Index System

Health information service refers to information organizations providing users with valuable health information in a specific way to meet their health needs and help them make healthy choices [29,30]. Xing Yao Zhang et al. designed the online health information service user satisfaction evaluation index system table with reference to four classical customer satisfaction theory evaluation models, including the Swedish SCSB (Sweden Customer Satisfaction Barometer) model [31], the American ACSI (American Customer Satisfaction Index) model [32], the European ECSI (European Customer Satisfaction Index) model [33], and the American public sector ASCI model, combined with a modified Chinese consumer behavior satisfaction CCSI (Chinese Customer Satisfaction Index) model [34,35,36], and structural equation modeling to analyze quantitatively in the empirical study. The concept of consumer satisfaction was first introduced in the field of marketing to measure the subjective evaluation of consumers during or after using a product or service [37]. The relevant scales and related materials referenced in this study include Internet health information rating systems, such as DISCERN [38,39] and the HON code.

## 3. Research Methodology

### 3.1. Construction of Evaluation Index System

Through the evaluation index system introduced in 2.3, this paper deletes, modifies, and completes the scale to form the criterion layer of a DXY satisfaction evaluation index, namely, perceived quality, perceived value, user trust, and user participation. The meanings of each variable and the evaluation specifics are described in detail in Table 1 with the characteristics of the health information service field. The details are as follows:

(1) Perceived quality refers to the user’s overall feeling of service quality after using DXY. By referring to the evaluation standards of online medical information resources by relevant organizations and researchers at home and abroad, starting from the quality standards of health information resources, combined with the characteristics of information services provided by the network, through user interviews, we found that users’ perception of quality can be divided into two aspects: platform information quality and platform performance quality. Platform information quality refers to the quality level of various types of information provided by DXY to users. The content of information service determines the technical factor; the quality of information content is directly related to the overall quality of information service. The perceived quality evaluation index is determined as authority Qa1, comprehensiveness Qc3, timeliness Qc2, effectiveness Qa2, professionalism Qb1, and easiness of information Qa3 [40,41,42,43].

**Table 1:** Satisfaction evaluation index system of DXY.

Guideline Layer	Program Level	Indicator Source
Q: Perceived quality	Qa1: Information authority	Nahapiet (2000) [44] Parasuraman et al. (2005) [45] Sabiote et al. (2012) [46] Sheng et al. (2010) [47] Yoo et al. (2001) [48] Barnes et al. (2002) [49] Barrera et al. (2014) [50] Barrutia et al. (2012) [51] Rolland et al. (2010) [52]
	Qa2: Information validity	
	Qa3: Information intelligibility	
	Qa4: Design aesthetics	
	Qb1: Information professionalism	
	Qb2: Question responsiveness	
	Qc1: Convenience of operation	
	Qc2: Information timeliness	
	Qc3: Information comprehensiveness	
	Qc4: Classification clarity	
V: Perceived value	Va1: Personalization	Magee et al. (2012) [11] Barnes et al. (2002) [49] Barrera et al. (2014) [50] Xinyao et al. (2010) [53]
	Va2: Privacy	
	Vb1: Time and expense cost	
	Vb3: Communication cost	
	Vc1: Science	
T: Users trust	Ta1: Development prospects	Barrutia et al. (2012) [51]
	Tb1: Utilization rate	
	Tc1: Recommendability	
P: User participation	Pa1: Online consultation	Tang et al. (2015) [36] Rolland et al. (2010) [52]
	Pa2: Willingness to pay to use	
	Pb1: Browse information frequency	
	Pb2: Online appointment willingness	
	Pc1: Interactive Discussion Willingness	

The strong interactivity of the Internet is one of its greatest advantages. Under the circumstance of limited functions, a perfect interactive experience is given to users as much as possible through a reasonable website content layout and a clear website layout with clear board levels. Information service provision can meet the personalized needs of users. The professionalism of the website navigation system is one of the important influencing factors to ensure users’ effective access to information, and to some extent, it also determines users’ feelings about using the website. Therefore, the evaluation of a health information service function consists of four indexes: problem response Qb2, design aesthetic Qa4, operation convenience Qc1, and classification clarity Qc4 [44,45,46,47,48,49,50,51,52].

(2) Perceived value refers to the cost of time, economy, expense, and energy spent by users when using DXY. Perceived value measures the degree of personal benefit to users when using health information services, and the evaluation of perceived value mainly includes saving time and money, meeting personalized needs, helping to communicate with doctors, and protecting personal privacy. The scheme-level indicators of perceived value include personalization Va1, privacy Va2 [11,53], time and expense cost Vb1, communication cost Vb3, and science Vc1 [49,50].

(3) User trust refers to the fact that users will develop trust in information services with an excessive degree of satisfaction after using them. On the one hand, they will continue to use different functions of online information services to satisfy their health needs, and on the other hand, they will promote and recommend to others to use the information service. User trust is the outcome variable, and user satisfaction is positively associated with the degree of trust users have in the information service. The solution-level indicators of user trust include development prospect Ta1, usage rate Tb1, and recommend ability Tc1 [51].

(4) User participation is the behavior of users who actively participate in activities related to information services based on their satisfaction and trust in the services after using the information services. User participation evaluation is mainly reflected by the frequency of users browsing online health information, using online doctors’ consultation service, online reservation and registration function, health interactive discussion, and paid use of the service. Therefore, user participation is the outcome variable, and users’ overall satisfaction with information services is positively correlated with user participation, and user trust is positively correlated with user participation. The solution-level indicators of user participation include willingness to consult online Pa1 [36], willingness to pay for use Pa2, frequency of browsing information Pb1, willingness to make an appointment online Pb2, and willingness to discuss interactively Pc1 [52].

### 3.2. Questionnaire Method

We use the questionnaire method to collect user usage data. Based on an extensive literature review on satisfaction with public health Internet platforms and information services on online health websites, a 23-question satisfaction questionnaire (Table 2) was finally developed to assess users’ satisfaction with DXY. The survey and research process was as follows: (1) Reading relevant literature. (2) Developing satisfaction evaluation indexes for DXY; the evaluation indexes were divided into four criterion levels of perceived quality, perceived value, user confidence, and user involvement, and corresponding scheme levels were designed under each criterion level, with a total of 23 scheme levels. The satisfaction evaluation is based on a 9-point scale, where points 1−9 indicate different degrees of satisfaction with DXY APP, where point 1 indicates extremely dissatisfied and point 9 indicates very satisfied. (3) The relevant questionnaires were designed according to the indicators, and web questionnaires were distributed and data collected.

A web-based questionnaire was provided through Questionnaire Star and Questionnaire.com to assess platform satisfaction. The first part of the questionnaire was for the personal information of the respondents, who were asked to be users who were using or had used the health platform of DXY and to evaluate their satisfaction with the index descriptions in Table 1. The questionnaire was in the form of a scale, and each question consisted of a set of statements. The questionnaire included basic information (gender, age, and education level and whether they had used DXY) and satisfaction with DXY (e.g., how aesthetically pleasing the platform’s page design is). User data were collected by distributing online questionnaires to users of DXY, and the questionnaires were distributed from June to August 2022. A total of 556 questionnaires were distributed, and 520 were collected, with a response rate of 93.53%, and 424 questionnaires remained after excluding some invalid questionnaires, with a validity rate of 81.54%.

### 3.3. Hierarchical Analysis and Entropy Method of Integrated Weighting

Hierarchical evaluation is a multicriteria decision-making technique proposed by using Thomas at the University of Pittsburgh in the early 1970s [54]. The method combines qualitative textual expression with quantitative numerical comparison and quantitative analysis as a guide and mathematical model as a tool, which can effectively avoid the subjective one-sidedness of demand transformation in the analysis and decision making of complex problems by systematizing and modeling a limited data sample [17]. Apply the hierarchical analysis method to calculate the weights of the criterion degree and the answer degree in the questionnaire records, respectively, and then the obtained results are calculated to derive the final weights of the hierarchical analysis method.

#### 3.3.1. Build a Comparison Judgment Matrix

AHP hierarchical evaluation is a technique to systematize complicated problems, and its fundamental concept is to set up a hierarchical shape mannequin of complicated choice troubles, compare and judge the evaluation indexes between two and two, make a comprehensive evaluation by quantification, and arrive at a rating of the relative significance of selection options [55]. The form of the evaluation matrix is as follows:

(1) $$R=\left(\begin{array}{cccc} r_{11} & r_{12} & \ldots & r_{1n}\\ r_{21} & r_{22} & \ldots & r_{2n}\\ \ldots & \ldots & \ldots & \ldots \\ r_{n1} & r_{n2} & \ldots & r_{nn} \end{array}\right)$$

The importance of $x_{i}$ relative to $x_{j}$ to r is indicated by $r_{ij}$, and the assigned value of $r_{ji}$ is usually assigned by relevant experts to assess the relative importance of indicators or given based on the data of the questionnaire survey, and has $r_{ij}\times r_{ji}=1$. Through the above judgment matrix, the weight price of every indicator is the eigenvector corresponding to the most eigenvalue of the judgment matrix, and the rectangular root technique is used to calculate the weight fee of the judgment matrix to derive the complete indicator weights for the pride comparison of DXY.

#### 3.3.2. Calculate the Weighting Factor

From the Perron–Frobenius theorem, we know that the matrix R has a unique nonzero eigenroot, namely, the largest eigenroot ($\lambda_{max}$) corresponding to the eigenvector (w):

(2) $$B_{w}=\lambda_{max}$$

The specific steps for calculating the feature vectors using the sum-product method are as follows:

Normalize the R data in the column normalized by:

(3) $$\tilde{r_{ij}}=\frac{r_{ij}}{\sum_{k=1}^{n}r_{kj}\left(i,j=1,2,\ldots ,n\right)}$$

Summing the normalized matrix peers.

(4) $$\tilde{w_{i}}=\sum_{j=1}^{n}\bar{r_{ij}}\left(i=1,2,\ldots ,n\right)$$

The weight vector is obtained by dividing the summed vector by *n*.

(5) $$\tilde{w_{i}}=\frac{\tilde{w_{i}}}{n}$$

Maximum characteristic root.

(6) $$\lambda_{\max}=\frac{1}{n}\overset{n}{\underset{i=1}{\sum }}\frac{{\left(R_{w}\right)}_{i}}{w_{i}}$$

where ${\left(R_{w}\right)}_{i}$ denotes the vector $R_{w}$ of the *i*-th component of the vector.

#### 3.3.3. Consistency Check

In order to stay away from the impact of subjective elements of the subjects, the consistency takes a look at the matrix R once carried out, and the check process used to be as follows:

(7) $$\mathrm{CR}=\frac{\mathrm{CI}}{\mathrm{RI}}$$

where CI is the consistency index, CR is the consistency ratio, and RI is the common random consistency index.

(8) $$\mathrm{CI}=\frac{\lambda_{\max}-n}{n-1}$$

CR can be calculated from Equations (7) to (8). Commonly, the smaller the CR value, the higher the consistency of the judgment matrix. Usually, when the value of CR is much less than 0.1, the judgment matrix satisfies the consistency test. If the value of CR is over 0.1, it means that there is no consistency, and the judgment matrix should be reanalyzed after appropriate adjustment.

In order to further achieve objectivity in the weight determination process, this study will use a combination of hierarchical analysis and entropy method to calculate the weights. Entropy is a notion in data theory, which is a measure of uncertainty. The larger the amount of information, the smaller the uncertainty, the smaller the entropy; the smaller the amount of information, the larger the uncertainty, the higher the entropy. According to the definition of statistical entropy, the entropy can be used to decide the degree of dispersion of a positive index; the smaller the entropy value is, the higher the degree of dispersion of the index is, and the higher the impact (i.e., weight) of the index on the overall assessment is. Therefore, the statistical entropy can be used to calculate the weights of each indicator, thus providing a basis for a complete comparison of several indicators. The combination of the two methods is helpful to improve the scientific weight of the DXY satisfaction evaluation index to ensure the accuracy of the final evaluation results.

Before calculating the entropy value method, the indicators should first be non-negative; there are no negative indicators in this study. The indicators are normalized, and the calculation formula is as follows:

(9) $$Y_{ij}=\frac{B_{ij}-{\left(B_{j}\right)}_{min}}{{\left(B_{j}\right)}_{max}-{\left(B_{i}\right)}_{min}}$$

$B_{ij}$ are the original data; *i* = 1, 2, 3, …, *m*; *j* = 1, 2, 3, …, *n*; and *i* and *j* denote the need of the evaluated unit and the number of evaluation indicators, respectively, and represent the maximum and minimum values in the *j* column of evaluation indicators, respectively. Since percentage variables are involved in the indicators, in order to avoid the case where the weight is 0, the indicators with a normalized value of 0 are uniformly calculated at 0.01.

First, calculate the weight of the *i* evaluation unit under the *j* indicator $P_{ij}$:

(10) $$P_{ij}=\frac{Y_{ij}}{\sum_{i=1}^{m}Y_{ij}}$$

Calculate the entropy value of the *j* metrics $e_{j}$:

(11) $$e_{j}=-\frac{1}{\ln n}\sum_{i=1}^{m}P_{ij}\ln P_{ij}$$

where $e_{j}$ is the entropy value of the *j*th indicator, *n* is the number of evaluation indicators, and ln is the natural logarithm function.

Calculate the entropy value of the *j* indicator $S_{j}$:

(12) $$S_{j}=\frac{1-e_{j}}{\sum_{j=1}^{n}1-e_{j}}$$

Based on the outcomes of the above two strategies of assigning weights to the symptoms, the combined weights are calculated $C_{j}$:

(13) $$C_{j}=\frac{{WS}_{j}}{\sum_{j=1}^{n}{WS}_{j}}$$

where *W* and $S_{j}$ represent the weights of evaluation indexes calculated by hierarchical analysis and the entropy value method, respectively.

## 4. Result

### 4.1. Reliability and Validity Tests

#### 4.1.1. Reliability Test

If the reliability coefficient of the scale is above 0.9, the reliability of the scale is excellent. If the coefficient is below 0.5, we need to reflect and reformulate the questionnaire. The reliability coefficient of the whole scale is ideally above 0.8, and between 0.7 and 0.8, it is acceptable. (The specific values of each item are shown in Table A1.)

The results showed (Table 3) that the value of Cronbach’s α coefficient was 0.978, indicating that the reliability of the questionnaire is relatively high.

#### 4.1.2. Validity Test

For the KMO test, a KMO value over 0.9 means that the scale is very suitable for analysis; between 0.8 and 0.9 is relatively suitable. For Bartlett’s test, if the value of *p* is much less than 0.05 or 0.01, the speculation is rejected. If the speculation is not rejected, this indicates that these variables can also independently provide some records and are now not appropriate for component evaluation. (The full validity test values are shown in Table A2.)

The consequences of KMO is 0.977, while the effects of Bartlett’s spherical test confirmed that the *p*-value was 0.000 *** (*p* < 0.001). The statistical significance level is reached, rejecting the speculation that there used to be correlation between the variables (Table 4).

### 4.2. Basic Characteristics of Survey Respondents

According to the statistics of the questionnaire survey (Table 5), the ratio of men to women is close to 1:1.88, indicating that women are more inclined to use DXY. In terms of age distribution, users aged 18 to 30 are the main force of DXY, followed by users aged 31 to 40, indicating that users of DXY are inclined to be younger. In terms of educational background, 59.91% of members have a bachelor’s degree or higher, with the majority of users having high education. Among those who have used DXY, most people use DXY to obtain health information, accounting for 43.87%; the number of people who use DXY for remote consultation is also high, reaching 40.33%; the number of people who purchase medicine online through DXY is the least, only 20.76%.

The survey results show that the age of users of DXY is concentrated in the young group of 18–30 years old, accounting for up to 70%, and influenced by gender and education; the usage rate of people with high education is higher, which is determined by the technical and knowledge attributes of the DXY software; and the usage rate of older and less educated people with relatively lower information literacy and restricted by personal ability is lower. The usage rate of the platform reflects that users tend to use the platform to learn about health information and expand their medical and health knowledge while working and studying, which do not require too much energy and financial resources; more users choose to use the remote consultation function of DXY, which is different from the traditional mode of seeing a doctor, that is, going to a physical hospital, and remote consultation can save the time and money cost of seeing a doctor. Remote consultation can save time and money and improve the convenience of daily visits to the doctor; at present, only a small number of users are willing to purchase medicine through the platform.

### 4.3. Indicator Weights Established

#### 4.3.1. Hierarchical Analysis Method to Determine the Index System Weights

In this study, after constructing and completing the hierarchical functional index system, we analyzed the user satisfaction indexes of DXY by distributing questionnaires and applying the AHP hierarchical evaluation approach to derive the weight values of each index and complete the consistency test.

In this study, the mean value of the analyzed items is calculated by default using SPSSAU (https://spssau.com, accessed on 23 October 2022), and the relative importance magnitude is obtained using the mean value information to assemble the judgment matrix wished for the AHP hierarchical evaluation to calculate the weights. Thus, the greater the number, the greater the relative importance. The judgment matrix is used to establish the relationship between the factors at each level in the form of a structural diagram and to compare the importance of all relevant factors within a certain step level between two. In this study, 556 questionnaires were distributed, 520 were returned, 424 were left after excluding some invalid questionnaires, and two-by-two comparisons were made for each evaluation index using a scale of 1 to 9. 

According to Equations (1)–(6), the criterion level weight values of satisfaction of DXY were calculated (Table 6), and the corresponding weight values of the four indicators were obtained from the analysis: 24.766%, 25.950%, 25.442%, and 23.842%. In addition, the most attribute root value (4.000) can be calculated by combining the function vectors, and then the CI value (0.000) is calculated by using the most attribute root value, which is used for the following consistency test. Based on the above ideas, the judgment matrix construction and weight calculation are performed for the satisfaction scheme layer of DXY.

According to Equations (7)–(8), the CI value calculated for the judgment matrix of the criterion layer is 0.000, and the RI value is 0.890, so the calculated CR is 0, less than 0.1, indicating that the judgment matrix of this scale passes the consistency test and the calculated weights are stable. Similarly, the CR values of the remaining judgment matrices are all 0, indicating that all the judgment matrices pass the consistency test (Table A3, Table A4, Table A5 and Table A6).

Using SPSSAU to calculate the weights of the program layer to which the four criterion layers belonged according to the above steps as well as the consistency test (Table 7, Table 8 and Table 9), it was concluded that the consistency evaluation of the judgment matrix all satisfied CR < 0.1 and passed the consistency test. From this, the scheme layer elements can be weighted and calculated to obtain the comprehensive weight value w of the scheme layer elements’ objectives (Table 7). Perceived quality, information ease of understanding Qa3, problem responsiveness Qb2, operational convenience Qc1, information timeliness Qc2, information comprehensiveness Qc3, and classification clarity Qc4 all have weight values of 10% or more. The perceived value dimensions of privacy Va2, time and cost Vb1, and communication cost Vb3 all have a weight value of more than 20%. The user trust dimension has a weighting of more than 30%, with the highest weighting of 34.168% for Ta1 on the development prospect of DXY. User participation dimension online consultation willingness Pa1 and browsing information frequency Pb1 both have weight values greater than 20%, 20.235%, and 21.308%, respectively. 

#### 4.3.2. Entropy Value Method to Determine the Index System Weights

The weighting coefficients of all elements can be received (Table 8). Using the entropy method to calculate the weights for a total of 23 items, such as Qa1, it can be seen that, Qa1, Qa2, Qa3, Qa4, Qb1, Qb2, Qc1, Qc2, Qc3, Qc4, Va1, Va2, Vb1, Vb3, Vc1, Ta1, Tb1, Tc1, Pa1, Pa2, Pb1, Pb2, and Pc1, a total of 23 items, their weight values are 0.051, 0.047, 0.038, 0.041, 0.042, 0.042, 0.042, 0.040, 0.034, 0.036, 0.041, 0.039, 0.038, 0.040, 0.039, 0.041, 0.045, 0.049, 0.048, and 0.067, respectively. The weight of each item was relatively even, all around 0.043.

#### 4.3.3. Combined Weights for the Combination of Hierarchical Analysis and the Entropy Method

According to Equation (13), the results of subjective distribution and objective distribution are calculated comprehensively (Table 9). A total of 23 items, such as weight Qa1, are calculated using the combination of the analytic hierarchy process and entropy method. It can be seen that, Qa1, Qa2, Qa3, Qa4, Qb1, Qb2, Qc1, Qc2, Qc3, Qc4, Va1, Va2, Vb1, Vb3, Vc1, Ta1, Tb1, Tc1, Pa1, Pa2, Pb1, Pb2, Pc1, a total of 23 items, their weight values are 2.687%, 2.505%, 2.209%, 2.209%, 2.368%, 2.460%, 2.368%, 2.255%, 2.027%, 2.141%, 4.691%, 4.828%, 4.464%, 4.714%, 4.555%, 8.107%, 8.677%, 9.337%, 5.284%, 6.787%, 4.259%, 5.466%, and 5.602%, respectively.

### 4.4. Data Analysis

The weight calculation of the questionnaire data of the criterion layer of the satisfaction evaluation system of DXY was performed by SPSSAU, and the corresponding weight values of a total of four items of perceived quality Q, perceived value V, user trust T, and user involvement P were obtained as 24.766%, 25.950%, 25.442%, and 23.842%, respectively. The weights were calculated separately for the scheme layers affiliated with the criterion layer, and the results were obtained: Qa1, Qa2, Qa3, Qa4, Qb1, Qb2, Qc1, Qc2, Qc3, and Qc4, in total 10 items corresponding to the weight values of 9.423%, 9.455%, 10.256%, 9.540%, 9.957%, 10.448%, 10.078%, and 10.078%. The corresponding weight values for Va1, Va2, Vb1, Vb3, and Vc1 are 19.304%, 20.785%, 20.079%, 20.011%, and 19.821% for a total of 5 items. The weights of Pa1, Pa2, Pb1, Pb2, and Pc1 are 20.235%, 18.704%, 21.308%, 19.969%, and 19.784%, respectively. The comprehensive weights calculated based on the AHP hierarchical analysis were then calculated by the weight values of the criterion and solution layers, and the results are shown in Appendix A.

In order to further improve the objectivity of the weight determination process, the entropy method is used again to calculate the weights of the indicators (Table 8). Finally, based on the weight values derived from the above two methods, the comprehensive weights were calculated by applying Equation 13 (Table 9). According to the results of the study, it is confirmed that the weighting of the indicators using the two methods is significantly different, and the weighting coefficients of the two methods are significantly different. This is due to the difference between the weights calculated by the mathematical model and the managers’ understanding of the application of the indicators in practice, which leads to the difference in the weighting coefficients, thus also further confirming the need for a study on the assignment of subjective and objective integrated weights.

**Figure 1:**
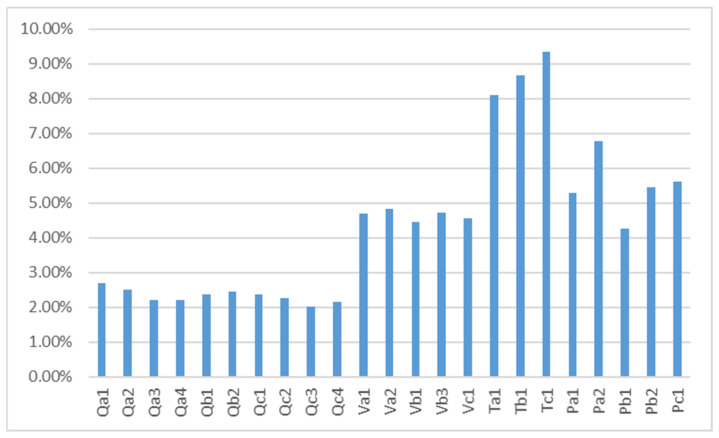
Combined weighting of indicators.

The final comprehensive weight value obtained by combining the two methods is established into a histogram (Figure 1). Through Figure 1, users’ satisfaction with the perceived quality of DXY is relatively low, all below 3%, with the lowest weight value of information comprehensiveness Qc3, only 2.027%, indicating that the health information released by DXY is not comprehensive enough to meet users’ expectations. The weight value of user trust is above 8%, which indicates that users are more optimistic about the future development prospect of DXY, have a higher possibility of increasing the usage rate of the platform in the future, and have the highest possibility of recommending the platform to their relatives and friends, which reflects that users are more satisfied with the information service provided by the current platform after using DXY, and have trust in DXY. 

The final weight of perceived value is relatively even, all between 4% and 5%, where users attach the greatest importance to the protection of personal privacy on the platform and are not yet satisfied with the current savings of time and money on the platform. According to the analysis of the graph, the highest weight is given to the user’s decision to use the paid services of DXY in the future, but the lowest weight is given to the frequency of browsing information on DXY in the future. This is the behavioral choice made by users after using the platform based on their satisfaction and trust in the information service of the platform and considering their future health needs and expectations of the platform.

According to the weighting results, at the perception level, users pay more attention to the content of perceived value, that is, the cost of time, economy, expense, and effort spent by users in using the DXY platform, emphasizing the degree of personal benefit. Overall, it is observed that users are less satisfied with the ease of understanding information Qa3, aesthetic design Qa4, timeliness of information Qc2, comprehensiveness of information Qc3, and clarity of classification Qc4 of DXY and more optimistic about the development prospects of the platform (Table 1).

## 5. Discussion and Suggestions

### 5.1. Perceived Quality Dimension

Perceived quality has a significant positive effect on DXY user satisfaction. Generally speaking, the reliability of the source of information resources, the authenticity of information disclosure content, advertising policy, website attributes, and other evaluation contents are important criteria to reflect the authority of information. Health information content should be comprehensive and closely related to the subject, such as the definition of disease, symptoms, treatment methods, treatment process, and other information for evaluation [54,55,56]. The information content material should be updated in a timely manner and must indicate the identity of the author of the published information; the statistics content material should be updated in a timely manner and must indicate the identity of the author of the published information, source, qualification, attribution, background, basis of information screening, sponsor information, the cause of publishing facts [57]. The stance of the information publisher should be objective and neutral without bias or implication, and the information itself should be easy to read and understand for nonmedical users.

Data evaluation indicates that customers are much less satisfied with the ease of grasp data Qa3, aesthetic design Qa4, timeliness of information Qc2, comprehensiveness of information Qc3, and clarity of classification Qc4 of DXY, with comprehensive weighting results of 2.209%, 2.209%, 2.255%, 2.027%, and 2.141%, respectively, so the platform should focus on improving the quality of offerings in these five components. In this regard, this paper puts forward the following recommendations in terms of both platform information quality and platform service quality.

(1) The information quality of the platform should improve the update frequency, increase the quantity of information, and optimize the information presentation. The platform can give attention to the current news and the timely release health science information in response to the current social health issues. For example, it should disseminate information on the principles of heat stroke and preventive measures to the public in response to the news of novel coronavirus and death from heat stroke in extreme hot weather. 

Based on meeting the needs of the general public, the platform functions and contents will be enriched for different user groups, and the health information of interest to users will be pushed precisely according to their habits, and the content of niche health information will be increased to reduce the risk of losing users due to a single content. The platform can also provide users with various forms of resources, such as pathology reports, research papers, and online lectures, by further mining and integrating all relevant information, such as pathogenesis, clinical manifestations, and cure cases, in the whole process of disease prevention, detection, and treatment. 

In terms of presenting health information, try to make complex medical knowledge easy to understand and popularize, so as to facilitate the understanding of information content by public users who lack medical background. It is also possible to expand the form of presentation of health information content and transform complex and difficult text into the form of video explanation, which helps users to receive information. 

Based on research on the basic characteristics of users in Table 5, it is found that among existing users of the platform, people of older age and lower education account for a minority, and the current users’ usage scenario is usually the daily leisure fragmented time to use DXY to obtain health information. The optimized information presentation can simultaneously take care of people of older age and lower education who are limited by their personal ability and their ability to understand information. 

In addition, popularized, interesting, and simplified content of the platform will increase users’ interest and reading quality and maintain the platform’s long-term attractiveness and competitiveness.

(2) In terms of platform performance quality, on the one hand, it is necessary to clearly divide different categories of information content, simplify the process of using DXY, and exclude the use of technical and equipment barriers. In this way, it can attract the public, especially the elderly users; enhance the sense of ease of use; improve the user experience; and further enhance the convenience of using the platform to improve user satisfaction. For example, in the website, prominently position to provide search engines, title, keywords, and other search methods.

On the other hand, improve the aesthetics of the platform interface design. A simple and beautiful interface design and a pleasing perception design can attract users visually, so platform developers can improve the interface and perception design of the platform with sufficient resources. Design various interfaces and features to meet customers’ personalized requirements, such as warm color and warm interface for female user groups, simplified operation interface for elderly groups, detailed version for user groups with medical background, and so on, to enhance the visual experience of users and attract a wider range of users.

### 5.2. Perceived Value Dimension

The perceived value dimension has a significant positive effect on DXY user satisfaction. The data analysis shows that the degree of influence of perceived value on user satisfaction is greater than that of perceived quality, and the combined weighting results are all greater than 4%. This indicates that users are more inclined to measure satisfaction in terms of value. According to the survey results, users attach the most importance to the platform’s protection of personal privacy in the perceived value dimension, and the combined weight result of privacy Va2 is 4.828%. Satisfaction in terms of saving time and cost is lower, with a combined weighting result of 4.464% for the time and expense cost Vb1 indicator. In response to this paper, the following recommendations are made:

(1) Improve privacy protection policies. With the improvement of the Internet, rising offerings such as social networks and the Internet of Things (IoT) have generated a wide variety of sorts of information at an unprecedented speed, ushering huge data. However, at the same time, how to protect nonpublic information and prevent the leakage of sensitive information has emerged as the most important task at present. Therefore, it is essential to first find out what data belong to the user’s personal information. The leakage of users’ personal information may affect their lives and the reputation of the platform, so the community must establish a more perfect privacy protection policy and clearly inform users when using it, and reduce users’ health privacy concerns through effective management methods and commitments, such as gradually opening more functional use to users by setting their growth mode in the platform. For patient consultation records, users can decide by themselves whether to disclose them and for how long.

(2) Improve the user’s interactive experience. This paper suggests that platform developers should improve the information retrieval methods and provide users with more diverse information retrieval methods as much as possible to enhance user convenience. At present, the information on the platform can only be searched by disease and specialty online and offline. It is suggested to add more types of search directions according to gender, age, body parts, occupational diseases, and so on. In addition, it is suggested that the platform adopt the combination of online and offline modes and improve the information of offline physical hospitals, including doctors’ qualifications, the direction of treatment they are good at, hospital location information, and so on, so as to facilitate users’ reference and comparison to save time and money costs.

### 5.3. User Trust Dimension

According to the survey data, users have a relatively high level of trust in the platform after using DXY; the program level indicators are higher than 8% both for the development prospect of the platform and for personal use and recommendation ability. User trust, as an outcome variable, reflects the high level of user satisfaction. User participation reflects the willingness of users to use the platform in the future, as well as the demand of users for the future development of the platform. According to the data, users are most likely to use the paid services of DXY, and the combined weighting result of the Pa2 indicator of willingness to pay for use is 6.787%. Therefore, the platform can adopt ways to simplify the process of purchasing drugs for users, improve the categories of drugs, and speed up delivery services to increase users’ willingness to use the platform to purchase drugs. Enhance the professionalism of medical personnel; strictly screen the service personnel, such as registered doctors and authors of scientific articles, to ensure that they have professional medical knowledge and a good professional ethical level and show users detailed information about doctors to enhance users’ trust and willingness to pay for treatment online. Additionally, the platform can improve the membership mechanism, use the point system, and set user levels and other ways to enhance user participation.

In addition, users’ willingness to interact and discuss online is also high, and the combined weighting result of the Pc1 indicator of willingness to interact and discuss is 5.602%. Therefore, it is recommended to strengthen the social attributes of the platform; add independent interfaces for users to make remarks, comments, and shares; establish links between users; and set certain reward mechanisms to promote active participation in comment sharing. Through an all-round social experience online, a community of strong relationship users will be precipitated from ordinary weak relationship users, thus realizing the word-of-mouth fission of users.

## 6. Conclusions

Taking DXY as an example, this paper constructed evaluation indexes and questionnaires from four dimensions of perceived quality, perceived value, user trust, and user participation, and collected 424 valid questionnaires. The comprehensive weight of the obtained questionnaire data was calculated by AHP and the entropy method. The results show that users’ satisfaction with DXY presents the following characteristics: (1) Users pay more attention to the content of perceived value, including the cost of time, economy, expense, and energy spent, and emphasize the degree of personal benefit. (2) Users have low satisfaction with the information accessibility, design aesthetics, information timeliness, information comprehensiveness, classification clarity, and other aspects of the DXY platform. (3) In the dimension of perceived value, users are most concerned about the platform’s protection of personal privacy. (4) In the dimension of time, users have a high degree of trust in the platform, a strong intention to use the platform in the future, and a high degree of recognition of online interactive discussion, willingness to pay, and paid service.

Based on the analysis of the weight of users’ satisfaction with DXY, corresponding strategies are further proposed on the basis of improving users’ sense of experience. First, the platform should improve the update frequency, increase the amount of information, and optimize the way of information presentation. Second, classify different types of information content clearly to improve the convenience of the platform and the aesthetics of the platform interface design. Third, improve privacy protection policies. Fourth, improve the user interaction experience. Fifth, improve the drug purchase procedures. Sixth, enhance the professionalism of medical personnel. Seventh, strengthen the social features of the platform.

This paper further extends the research in related fields and enriches the research results. It also makes some contributions in terms of methodology, theory, and practical application, including the following three aspects: First, from the perspective of methodology, this paper further explores user satisfaction with an online medical platform based on DXY, and collects data by constructing an evaluation index system and questionnaire. AHP and the entropy method are used to calculate the weight. The combination of the two methods explores the allocation of subjective and objective comprehensive weights, further realizes the objectivity of the weight determination process, and forms a data analysis framework for the study of user satisfaction. Second, from the theoretical point of view, it further explores the research on user satisfaction with the public health Internet platform; emphasizes the influence of three dimensions of perceived quality, perceived value, and user trust on user satisfaction; and puts forward the promotion strategy of DXY through the dimension of user participation. Third, from the perspective of practical application, under the dual thrust of Internet development and global epidemic, the usage rate of an Internet medical platform has increased significantly. This study provides powerful data support and improvement strategies for users to improve their sense of use of an Internet medical platform, which is conducive to the improvement of an Internet medical platform.

However, it is also important to note that the current study has certain limitations. First, the group surveyed in this paper is limited. The surveyed users are not representative of all platform users. Data bias needs to be overcome in the future. The data in this study may omit some social groups, such as children and the elderly. In the future, we hope to collect more user questionnaires and improve the user data. Second, the scope of this study is limited to electronic participation. The questionnaire data in this paper are all from online questionnaires, and future research may focus on offline participation. Third, there has been no further analysis of specific user comments. Subsequent research work can be analyzed from the aspects of comment content identification and comment content clustering, and the research results can provide targeted help for the platform to improve the existing functions.

## Figures and Tables

**Table 2:** Description of the conversion questionnaire of the satisfaction evaluation index of DXY.

Program Level	Problem Description
Qa1: Information authority	The health information published on the platform has clear contact information for you
Qa2: Information validity	Health information release is effective for your treatment of diseases
Qa3: Information Intelligibility	The health information is written in an easy-to-understand way for you
Qa4: Design aesthetics	The aesthetics of the platform page design is important to you
Qb1: Information professionalism	Health information involves multidisciplinary outcomes for you
Qb2: Question responsiveness	Asking a health question can be responded to quickly for you
Qc1: Convenience of operation	The ability to open quickly on different types of devices for you
Qc2: Information timeliness	How often the platform health information is updated for you
Qc3: Information comprehensiveness	The comprehensiveness of the content of the health information is important to you
Qc4: Classification clarity	The information directory is clearly categorized for you
Va1: Personalization	The platform can meet your individual needs for you
Va2: Privacy	The platform can protect your personal privacy for you
Vb1: Time and expense cost	The platform can save you time and money costs for you
Vb3: Communication cost	The platform can help you to communicate with your doctor for you
Vc1: Science	The platform can enrich health knowledge and raise health awareness for you
Ta1: Development prospects	Are you optimistic about the future of Dr. Ding Xiang
Tb1: Utilization rate	The likelihood that you will increase your usage of Dr. Ding Xiang
Tc1: Recommend ability	How likely would you be to recommend Dr. Ding to your friends and family
Pa1: Online consultation will	The possibility of you using Dr. Ding’s online consultation service
Pa2: Willingness to pay to use	The possibility of you using the paid services of Dr. Ding
Pb1: Browse information frequency	Likelihood of you using Dr. Ding to browse health information
Pb2: Online appointment willingness	The possibility for you to use the online appointment service of Dr. Ding Xiang
Pc1: Interactive discussion willingness	Possibilities for you to participate in interactive health discussions using Dr. Ding

**Table 3:** Cronbach’s reliability analysis.

Cronbach’s Alpha Coefficient	Standardized Cronbach’s Alpha Coefficient	Number of Items	Number of Samples
0.978	0.978	24	424

**Table 4:** KMO test and Bartlett’s test.

KMO Value		0.977
Bartlett’s sphericity test	Approximate cardinality	10,499.405
	df	276.000
	*p*	0.000 ***

Note: *** represents a significance level of 1%.

**Table 5:** Basic characteristics of survey respondents.

Survey Object Attributes	Options	Number of People	Percentage (%)
Gender	Female	277	65.330
	Male	147	34.670
Age	18–30	297	70.047
	31–40	74	17.453
	Under 18 years old	26	6.132
	41–50	23	5.425
	51 or more	4	0.943
Academic qualifications	University	254	59.906
	Graduate students	105	24.764
	High school	49	11.557
	Junior high school and below	16	3.774
What features of DXY have you used?	Online registration	144	33.962
	Online payment	130	30.660
	Teleconsultation	171	40.330
	Inquiry report	100	23.585
	Online drug purchase	88	20.755
	Science and health information	186	43.868
	Other	60	14.151

**Table 6:** Criterion-level judgment matrix and weight values for satisfaction with DXY.

Item	Q	V	T	P	Eigenvector	Weighting Value	Maximum Eigenvalue	CI Value	CR Value
Q	1	0.954	0.973	1.039	0.991	24.766%	4.000	0.000	0.000
V	1.048	1	1.020	1.088	1.038	25.950%			
T	1.027	0.980	1	1.067	1.018	25.442%			
P	0.963	0.919	0.937	1	0.954	23.842%			

**Table 7:** Summary of the results of calculating comprehensive weights based on hierarchical analysis.

Target Layer	Guideline Layer	Weighting Value	Program Level	Weighting Value	Combined Weight *w*
Comprehensive weighting of satisfaction evaluation indicators of DXY	Q	24.766%	Qa1	9.423%	2.334%
			Qa2	9.455%	2.342%
			Qa3	10.256%	2.540%
			Qa4	9.540%	2.363%
			Qb1	9.957%	2.466%
			Qb2	10.448%	2.588%
			Qc1	10.078%	2.496%
			Qc2	10.014%	2.480%
			Qc3	10.430%	2.583%
			Qc4	10.398%	2.575%
	V	25.950%	Va1	19.304%	5.009%
			Va2	20.785%	5.394%
			Vb1	20.079%	5.211%
			Vb3	20.011%	5.193%
			Vc1	19.821%	5.144%
	T	25.442%	Ta1	34.168%	8.693%
			Tb1	33.036%	8.405%
			Tc1	32.796%	8.344%
	P	23.842%	Pa1	20.235%	4.824%
			Pa2	18.704%	4.459%
			Pb1	21.308%	5.080%
			Pb2	19.969%	4.761%
			Pc1	19.784%	4.717%

**Table 8:** Summary of the results of calculating weights based on the entropy value method.

Indicators	Information Entropy Value *e*	Information Utility Value *d*	$\mathrm{Weighting}\;\mathrm{Factor}\;S_{j}$
Qa1	0.9898	0.0102	5.07%
Qa2	0.9905	0.0095	4.70%
Qa3	0.9923	0.0077	3.82%
Qa4	0.9917	0.0083	4.11%
Qb1	0.9915	0.0085	4.20%
Qb2	0.9916	0.0084	4.17%
Qc1	0.9916	0.0084	4.18%
Qc2	0.9919	0.0081	4.01%
Qc3	0.9930	0.0070	3.45%
Qc4	0.9926	0.0074	3.65%
Va1	0.9917	0.0083	4.11%
Va2	0.9921	0.0079	3.93%
Vb1	0.9924	0.0076	3.77%
Vb3	0.9920	0.0080	3.98%
Vc1	0.9921	0.0079	3.89%
Ta1	0.9917	0.0083	4.09%
Tb1	0.9908	0.0092	4.53%
Tc1	0.9901	0.0099	4.91%
Pa1	0.9903	0.0097	4.80%
Pa2	0.9865	0.0135	6.68%
Pb1	0.9926	0.0074	3.68%
Pb2	0.9898	0.0102	5.05%
Pc1	0.9894	0.0106	5.22%

**Table 9:** Combined weights of the combination of hierarchical analysis and the entropy method.

Indicators	Hierarchical Analysis Method Weight *w*	$\mathrm{Entropy}\;\mathrm{Method}\;\mathrm{Weights}\;S_{j}$	$\mathrm{Combined}\;\mathrm{Weights}\;C_{j}$
Qa1	2.334%	5.07%	2.687%
Qa2	2.342%	4.70%	2.505%
Qa3	2.540%	3.82%	2.209%
Qa4	2.363%	4.11%	2.209%
Qb1	2.466%	4.20%	2.368%
Qb2	2.588%	4.17%	2.460%
Qc1	2.496%	4.18%	2.368%
Qc2	2.480%	4.01%	2.255%
Qc3	2.583%	3.45%	2.027%
Qc4	2.575%	3.65%	2.141%
Va1	5.009%	4.11%	4.691%
Va2	5.394%	3.93%	4.828%
Vb1	5.211%	3.77%	4.464%
Vb3	5.193%	3.98%	4.714%
Vc1	5.144%	3.89%	4.555%
Ta1	8.693%	4.09%	8.107%
Tb1	8.405%	4.53%	8.677%
Tc1	8.344%	4.91%	9.337%
Pa1	4.824%	4.80%	5.284%
Pa2	4.459%	6.68%	6.787%
Pb1	5.080%	3.68%	4.259%
Pb2	4.761%	5.05%	5.466%
Pc1	4.717%	5.22%	5.602%

## Data Availability

The experimental data used to support the findings of this study are included in the article.

## Appendix A

**Table A1:** Cronbach’s reliability analysis.

Indicators	Total Correlation of Correction Items	Alpha Coefficient of the Term Has Been Deleted	Cronbach’s Alpha Coefficient
Qa1	0.775	0.977	0.978
Qa2	0.804	0.977	
Qa3	0.798	0.977	
Qa4	0.725	0.977	
Qb1	0.779	0.977	
Qb2	0.809	0.977	
Qc1	0.802	0.977	
Qc2	0.807	0.977	
Qc3	0.834	0.976	
Qc4	0.835	0.976	
Va1	0.799	0.977	
Va2	0.789	0.977	
Vb1	0.786	0.977	
Vb3	0.817	0.976	
Vc1	0.828	0.976	
Ta1	0.830	0.976	
Tb1	0.783	0.977	
Tc1	0.802	0.977	
Pa1	0.794	0.977	
Pa2	0.693	0.977	
Pb1	0.831	0.976	
Pb2	0.780	0.977	
Pc1	0.741	0.977	

**Table A2:** Results of validity analysis.

Indicators	Factor Loading Coefficient		Common Degree (Variance of Common Factor)
	Factor 1	Factor 2	
Qa1	0.641	0.472	0.634
Qa2	0.663	0.488	0.679
Qa3	0.779	0.345	0.726
Qa4	0.594	0.455	0.560
Qb1	0.685	0.429	0.653
Qb2	0.797	0.339	0.750
Qc1	0.744	0.391	0.707
Qc2	0.668	0.489	0.685
Qc3	0.801	0.372	0.779
Qc4	0.823	0.346	0.798
Va1	0.723	0.412	0.693
Va2	0.832	0.270	0.765
Vb1	0.798	0.306	0.730
Vb3	0.726	0.435	0.716
Vc1	0.732	0.444	0.732
Ta1	0.559	0.650	0.734
Tb1	0.435	0.727	0.718
Tc1	0.388	0.809	0.805
Pa1	0.412	0.768	0.760
Pa2	0.224	0.843	0.760
Pb1	0.555	0.656	0.739
Pb2	0.390	0.772	0.748
Pc1	0.319	0.802	0.744
Characteristic root value (before rotation)	15.921	1.437	-
Variance interpretation rate % (before rotation)	66.336%	5.989%	-
Cumulative variance interpretation rate % (before rotation)	66.336%	72.325%	-
Characteristic root value (after rotation)	9.958	7.400	-
Variance interpretation rate % (after rotation)	41.492%	30.834%	-
Cumulative variance interpretation rate % (after rotation)	41.492%	72.325%	-
KMO value	0.977		-
Bartlett’s sphericity test	10,499.405		-
df	276		-
p	0.000		-

**Table A3:** Judgment matrix and weight value for “perceived quality”.

Item	Qa1	Qa2	Qa3	Qa4	Qb1	Qb2	Qc1	Qc2	Qc3	Qc4	Eigenvector	Weighting Value	Maximum Eigenvalue	CI Value	CR Value
Qa1	1	0.997	0.919	0.988	0.946	0.902	0.935	0.941	0.903	0.906	0.942	9.423%	10.000	0.000	0.000
Qa2	1.003	1	0.922	0.991	0.950	0.905	0.938	0.944	0.906	0.909	0.945	9.455%			
Qa3	1.088	1.085	1	1.075	1.030	0.982	1.018	1.024	0.983	0.986	1.026	10.256%			
Qa4	1.012	1.009	0.930	1	0.958	0.913	0.947	0.953	0.915	0.917	0.954	9.540%			
Qb1	1.057	1.053	0.971	1.044	1	0.953	0.988	0.994	0.955	0.958	0.996	9.957%			
Qb2	1.109	1.105	1.019	1.095	1.049	1	1.037	1.043	1.002	1.005	1.045	10.448%			
Qc1	1.070	1.066	0.983	1.056	1.012	0.965	1	1.006	0.966	0.969	1.008	10.078%			
Qc2	1.063	1.059	0.976	1.050	1.006	0.958	0.994	1	0.960	0.963	1.001	10.014%			
Qc3	1.107	1.103	1.017	1.093	1.048	0.998	1.035	1.042	1	1.003	1.043	10.430%			
Qc4	1.104	1.100	1.014	1.090	1.044	0.995	1.032	1.038	0.997	1	1.040	10.398%			

**Table A4:** Judgment matrix and weight value for “perceived value”.

Item	Va1	Va2	Vb1	Vb3	Vc1	Eigenvector	Weighting Value	Maximum Eigenvalue	CI Value	CR Value
Va1	1	0.929	0.961	0.965	0.974	0.965	19.304%	5.000	0.000	0.000
Va2	1.077	1	1.035	1.039	1.049	1.039	20.785%			
Vb1	1.040	0.966	1	1.003	1.013	1.004	20.079%			
Vb3	1.037	0.963	0.997	1	1.010	1.001	20.011%			
Vc1	1.027	0.954	0.987	0.990	1	0.991	19.821%			

**Table A5:** Judgment matrix and weight value for “user trust”.

Item	Ta1	Tb1	Tc1	Eigenvector	Weighting Value	Maximum Eigenvalue	CI Value	CR Value
Ta1	1	1.034	1.042	1.025	34.168%	3.000	0.000	0.000
Tb1	0.967	1	1.007	0.991	33.036%			
Tc1	0.960	0.993	1	0.984	32.796%			

**Table A6:** Judgment matrix and weight value for “user involvement”.

Item	Pa1	Pa2	Pb1	Pb2	Pc1	Eigenvector	Weighting Value	Maximum Eigenvalue	CI Value	CR Value
Pa1	1	1.082	0.950	1.013	1.023	1.012	20.235%	5.000	0.000	0.000
Pa2	0.924	1	0.878	0.937	0.945	0.935	18.704%			
Pb1	1.053	1.139	1	1.067	1.077	1.065	21.308%			
Pb2	0.987	1.068	0.937	1	1.009	0.998	19.969%			
Pc1	0.978	1.058	0.928	0.991	1	0.989	19.784%			
